# Prognostic Role of *FGFR3* Expression Status and Tumor-Related MicroRNAs Level in Association with PD-L1 Expression in Primary Luminal Non-Muscular Invasive Bladder Carcinoma

**DOI:** 10.3390/life10110305

**Published:** 2020-11-23

**Authors:** Ekaterina Blinova, Anton Buzdin, Dmitry Enikeev, Dmitry Roshchin, Maria Suntsova, Elena Samyshina, Aleksey Drobyshev, Olga Deryabina, Tatiana Demura, Dmitry Blinov, Evgenia Shich, Haydar Barakat, Pieter Borger, Dmitrij Merinov, Aleksandr Kachmazov, Stanislav Serebrianyi, Oxana Tumutolova, Natalia Potoldykova, Pavel Zhdanov, Vagarshak Grigoryan, Dmitrij Perepechin

**Affiliations:** 1 Department of Clinical Anatomy and Operative Surgery, Department of Pathological Anatomy, Institute for Urology and Reproductive Health, Sechenov University, 119991 Moscow, Russia; bev-sechenov@mail.ru (E.B.); dvenikeev@gmail.com (D.E.); demura-t@yandex.ru (T.D.); chih@mail.ru (E.S.); potoldykovanv@gmail.com (N.P.); zhdanov-pn@yandex.ru (P.Z.); nii-uronephrology@yandex.ru (V.G.); 2 Laboratory of Bioinformatics, Institute for Personalized Medicine, Sechenov University, 119991 Moscow, Russia; buzdin@oncobox.com (A.B.); suntsova@oncobox.com (M.S.); drobyshev@oncobox.com (A.D.); 3 Shemyakin-Ovchinnikov Institute of Bioorganic Chemistry, Russian Academy of Sciences, 117997 Moscow, Russia; 4 Russian National Research Center of Radiology, Department of Oncological Urology, 125284 Moscow, Russia; dr89031990702@gmail.com (D.R.); d.merinov@gmail.com (D.M.); kac.68@mail.ru (A.K.); volecon@mail.ru (S.S.); medcraft@mail.ru (D.P.); 5 All-Union Research Center for Biological Active Compounds Safety, Laboratory of Molecular Pharmacology and Drug Design, 142450 Staraja Kupavna, Russia; samy-elena@yandex.ru; 6 Laboratory of Pharmacology, Department of Oncology, National Research Ogarev Mordovia State University, 430005 Saransk, Russia; dep-general@adm.mrsu.ru (O.D.); tumutol@rambler.ru (O.T.); 7 Department of Propaedeutics of Dental Diseases, People’s Friendship University of Russia, 117198 Moscow, Russia; barakat-kh@rudn.ru; 8 Laboratory of the Swiss Hepato-Pancreato-Biliary and Transplantation Center, Department of Surgery, University Hospital Zürich, 8091 Zürich, Switzerland; pieter.borger@usz.ch

**Keywords:** non-muscular invasive bladder carcinoma, fibroblast growth factor receptor 3, mutation, prognosis, cancer progression, PD-L1, microRNA, expression, tumor relapse

## Abstract

Background: bladder cancer is one of the most common urinary tract malignancies. Establishment of robust predictors of disease progression and outcome is important for personalizing treatment of non-muscular invasive bladder carcinoma (NMIBC). In this study we evaluated association of PD-L1 expression with other prognostic biomarkers, such as expression of miRNA-145 and miRNA-200a, *FGFR3* gene expression, and mutation status in tissue specimens of the luminal subtype of newly diagnosed high and low grade NMIBC. Methods: twenty patients with primary luminal NMIBC were enrolled in the study. Tumor grade and risk level were determined in accordance with European Organization for Research and Treatment of Cancer (EORTC) guidelines and World Health Organization (WHO) classification. Neoplasm molecular subtype and PD-L1 expression level were assessed by immunohistochemistry. We used real-time PCR to evaluate the expression of microRNAs and *FGFR3*. We detected *FGFR3* hotspot mutations in codons 248 and 249 by Sanger sequencing. Results: high grade primary luminal NMIBC showed comparatively higher expression of PD-L1 and microRNA-145 than a low grade tumor, whereas the latter had a higher *FGFR3* expression and hotspot mutation rate. The tumor grade (HR = 571.72 [11.03–2.96] *p* = 0.002), PD-L1 expression (HR = 2.33 [0.92–1.92] *p* = 0.012), and *FGFR3* expression (HR = 0.08 [0.17–0.42] *p* = 0.003) were associated with relapse-free survival. Conclusions: tumor grade in association with PD-L1 and *FGFR3* expression can be considered as a complex predictor for primary luminal NMIBC progression.

## 1. Introduction

Bladder cancer remains to be one of the most common human genitourinary tumors worldwide with high cost per patient and high mortality rate [1,2]. Primary morphological features along with involvement of the muscular layer altogether determine two essential divergent pathways of bladder carcinoma development as non-muscular invasive bladder cancer (NMIBC) and muscular invasive bladder carcinoma (MIBC). The first one at the time of diagnosis was proven to be less aggressive than the latter [3,4], but treatment of both requires stringent interventional and surveillance protocols [5]. At the same time, biological and genetic heterogeneity of NMIBC serves as a basis for the disease molecular classification. According to the tumor molecular profiling made by Dadhania and colleagues, CR5/6 and GATA3 expression levels are currently used to designate NMIBC into basal, luminal, and double-negative subtypes [6].

Identification of the pivotal role of immune checkpoint molecules has drastically changed diagnostic, prognostic, and treatment approaches to many malignancies including bladder cancer [7,8,9]. Besides, different laboratories showed that expression levels of the programmed death (PD-1) receptor and its ligand 1 (PD-L1) may broadly vary depending on NMIBC grade and molecular subtype [10]. Moreover, check-point inhibitors’ clinical success closely relates to the tumor immune microenvironment [11], while differences in relapse-free survival (RFS) of patients with luminal, basal, and double-negative NMIBC of high- and low-malignant grade are strictly associated with previously utilized treatment [12].

MicroRNAs are small non-coding molecules involved in different regulatory functions and intracellular signaling [13,14]. Numerous studies have demonstrated prognostic role of microRNAs for NMIBC outcomes not only in tumor tissue-associated form, but also as stable cell-free molecules circulating in different biological fluids [15,16,17]. Yun et al. previously demonstrated an association between NMIBC diagnosis and progression and concentrations of microRNA-145 and microRNA-200a molecules [17]. These and other microRNAs influence tumor progression via the regulation of gene activities, including expression of fibroblast growth factor receptor 3 gene (*FGFR3*) [18,19]. *FGFR3* expression level and mutation status were shown to be determinants of bladder carcinoma progression [20].

In our previous study, we found that PD-L1 expression status was associated with RFS for luminal NMIBCs in the group without previous frontline intervention, and with RFS in the group of patients with luminal relapsed bladder cancer who previously used Bacillus Calmette-Guerin (BCG) [12]. We investigated here the prognostic roles of microRNA-200a, microRNA-145, *FGFR3* expression, and mutation status in association with PD-L1 expression in patients with primary luminal NMIBC.

## 2. Results

### 2.1. PD-L1 Expression in Primary Luminal NMIBC

**Figure 1:**
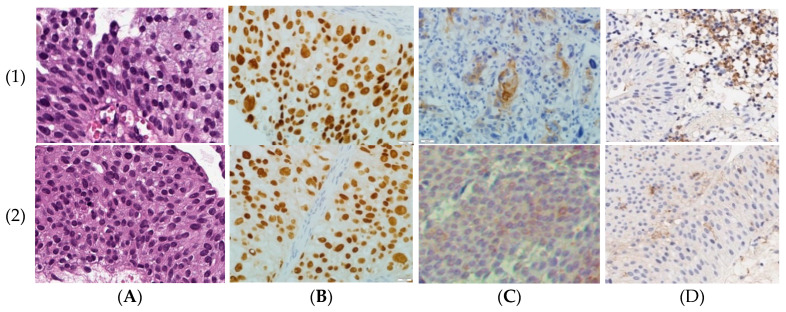
Morphological characteristic of tumors: (1) high grade primary luminal non-muscular invasive bladder cancer (NMIBC); (2) low grade primary luminal NMIBC. (**A**) Hematoxylin and eosin staining, ×500. (**B**) Anti-GATA3 IHC-staining, ×500. (**C**) Anti-CR5/6 IHC-staining, ×500. (**D**) Anti-PD-L1 IHC-staining, ×500.

**Figure 2:**
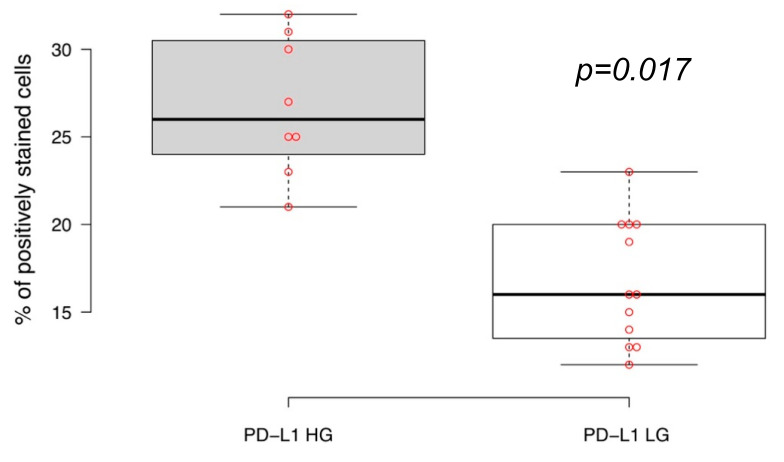
High-grade (HG) and low grade (LG) non-muscular invasive bladder cancers of luminal molecular subtype express PD-L1 differently. Boxplots show medians of % of tumor cellular membranes positively stained by anti-PD-L1 antibody (Ventana PD-L1 Assay (SP263)); significance of expression differences were estimated by independent *t* test.

We immunohistochemically assessed PD-L1 expression status of the luminal subtype of non-muscular invasive urothelial carcinomas with different tumor grades (Figure 1 and Figure 2). The proportion of cancer cells positively stained for the PD-L1 membrane expression was statistically higher in GATA3-positive high grade primary NMIBCs (n = 8) than in low grade bladder carcinomas (n = 12), with average values of 26.8% vs. 16.3%, respectively (*p* = 0.017).

### 2.2. FGFR3 Expression and Hotspot Mutations in Primary Luminal NMIBC

**Figure 3:**
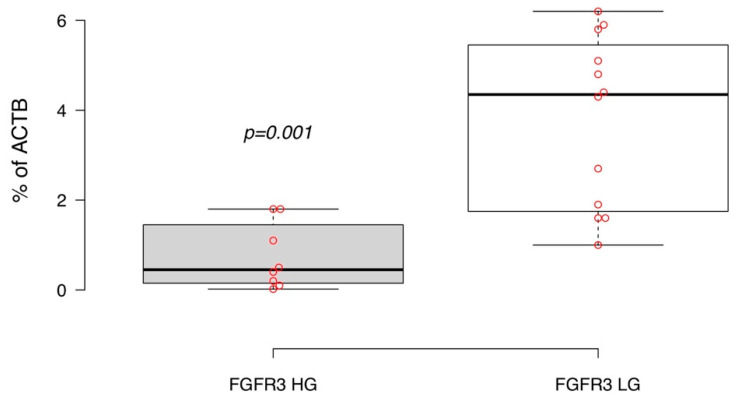
*FGFR3* expression level in primary diagnosed high (HG) and low grade (LG) luminal NMIBC. Boxplots display medians of % of beta-actin gene (ACTB) expression; significance of expression differences were estimated by independent *t* test.

**Figure A1:**
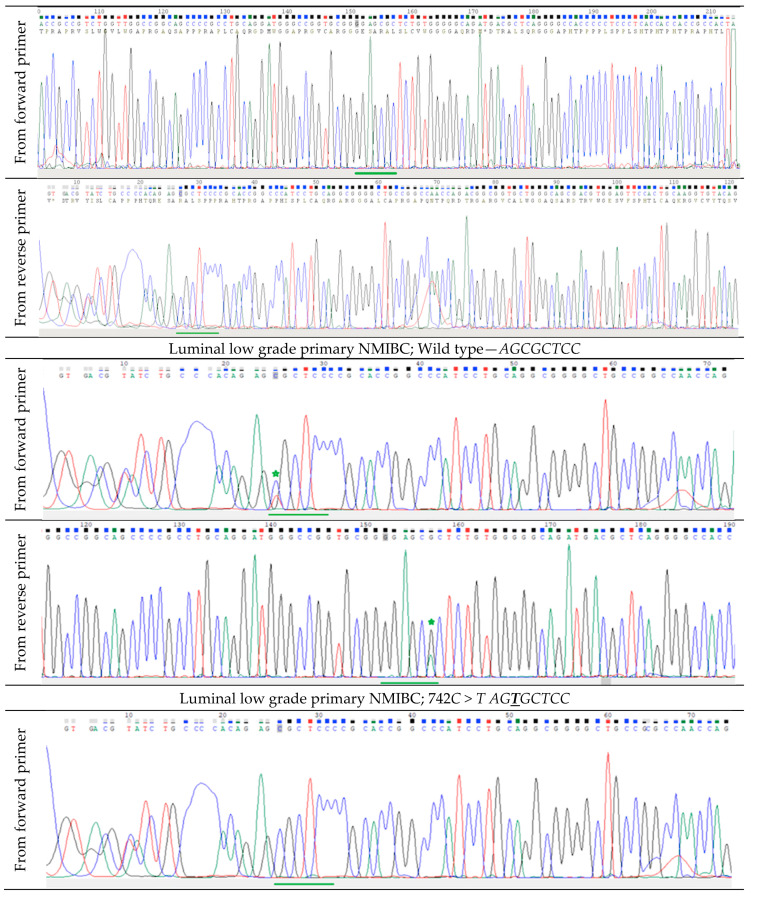

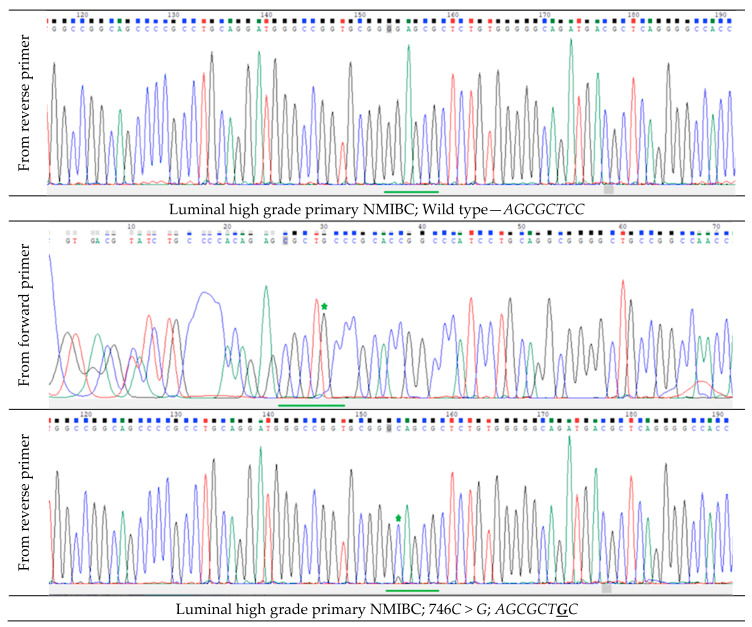
*FGFR3* gene hotspot mutations in codons 248 and 249: wild type allele is represented by *AG**C**GCT**C**C* nucleotide sequence; mutant genotypes had substitutions *C -> T* in position 3 or *C -> G* in position 7.

We then assessed the *FGFR3* gene transcript expression as the percentage of the *ACTB* housekeeping gene mRNA concentration. In our experiments, high grade luminal NMIBC was associated with relatively lower cellular expression of *FGFR3* compared to low grade non-invasive urothelial cancer (Figure 3, *p* = 0.001). Detecting *FGFR3* hotspot mutations by Sanger sequencing for both grades of primary luminal non-invasive bladder cancer also demonstrated the prevalence of the gene mutation rate in codons 248 and 249 in low grade tumor cells (66.6% of samples had *FGFR3* mutations), whereas this proportion was only 25% in high grade luminal NMIBCs (Figure A1). Interestingly, *FGFR3* mutation status was not correlated with this gene expression level.

### 2.3. MicroRNA-145 and MicroRNA-200a Expression in Primary Luminal NMIBC

**Figure 4:**
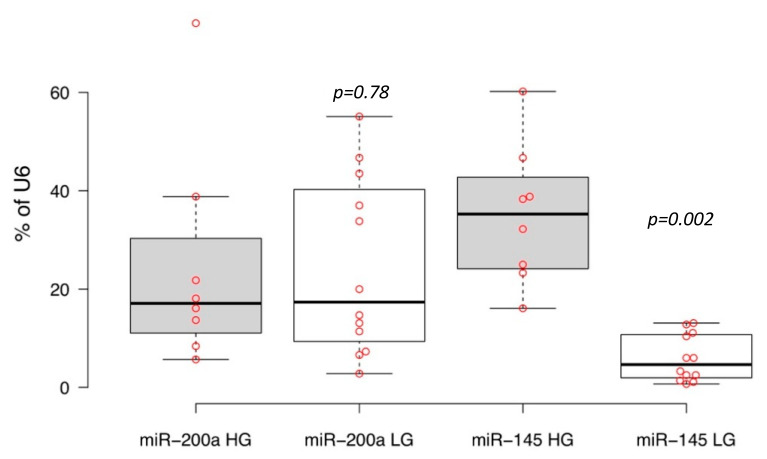
Expression of microRNA-200a (miR-200a) and microRNA-145 (miR-145) in primary high (HG) and low grade (LG) non-muscular invasive urothelial carcinoma of luminal molecular subtype. Boxplots display medians of percentage of U6 snRNA expression; significance of expression differences were estimated by independent *t* test.

We then assessed expressions of microRNAs-200a and -145 in the tumor samples under investigation by quantitative real-time reverse transcription polymerase chain reaction (PCR). The principle of the microRNA detection method used is described by Chen and co-authors (2005) [21]. This method is based on the individual reverse transcription of mature microRNA using a unique long stem-loop primer. The resulting product was detected by quantitative PCR with a specific fluorescent probe. MicroRNA levels were measured as the percentage of housekeeping U6 small nucleolar RNA expression. We found no statistically significant differences (*p* = 0.78) between more and less aggressive primary luminal subtypes of bladder cancer for cellular microRNA-200a expression levels; in contrast, the high grade luminal NMIBC tumor tissues had a ~5.9 times higher expression of microRNA-145 than low grade primary tumors (*p* = 0.002) (Figure 4).

### 2.4. PD-L1, FGFR3 and MicroRNAs Expression, and Relapse-Free Survival in Luminal NMIBC

**Figure 5:**
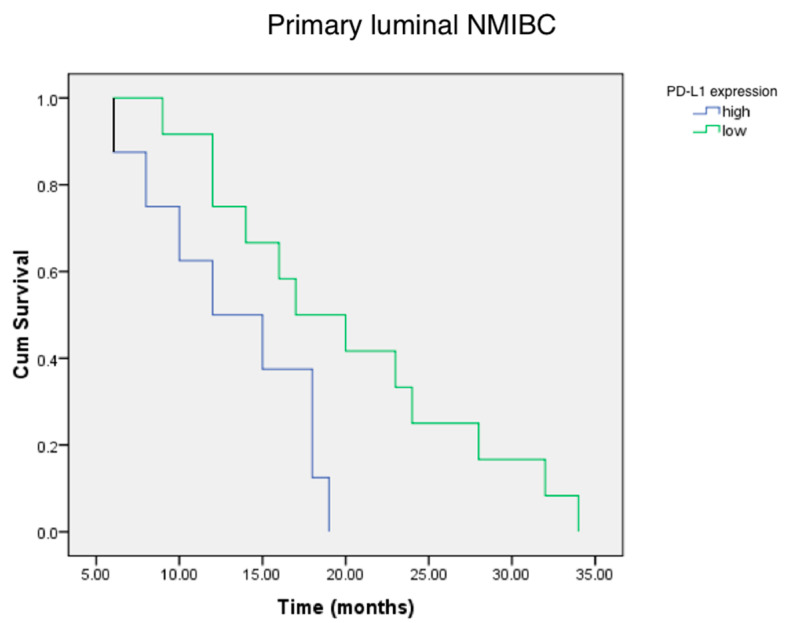
Cumulative survival (Cum Survival) of patients with high and low PD-L1 expressing the status of primary luminal NMIBC. According to long rank test, there is significant evidence of a difference in relapse times for low and high PD-L1+ status groups (*p* < 0.05).

Using the Kaplan–Meier actuarial analysis, we demonstrated that the time to first tumor relapse was lower for the patients with a high PD-L1 status of luminal primary cancer in comparison with less aggressive low PD-L1 expressing luminal NMIBC (Figure 5).

Using univariable analysis, we then found that the tumor grade (HR = 571.72 [11.03–2.96] *p* = 0.002), PD-L1 expression (HR = 2.33 [0.92–1.92] *p* = 0.012), and *FGFR3* expression (HR = 0.08 [0.17–0.42] *p* = 0.003) were significantly associated with relapse-free survival (Table 1). At the same time, there were no significant link between RFS and miR-145, mir-200a, and *FGFR3* gene mutation rate.

## 3. Discussion

Temporary cancer molecular biology delivers a broad range of novel molecules and markers that frequently play pivotal roles at some definite stages of tumor development and progression. From a clinician’s point of view, though, it is of great importance to base effective and safe treatment strategies on strong, proven, and, simultaneously and desirably, a limited panel of predictors, which reflects involvement of key pathways in the malignant process. Such internal ambiguity between galloping molecular science and quite conservative clinical medicine requires nontrivial approaches to the issue of the prediction of cancer progression. There is a wide panel of markers with different predictive values for the prognosis of bladder cancer behavior [22,23,24]. During recent decades, we observed how high expert boards tried to combine different clinical hallmarks of the disease and implement molecular novels for guidelines, consensuses, and classifications. As a result, sophisticated actual molecular and clinical classification of non-muscular-invasive bladder cancer illuminated an urgent need for stringent and reliable prognosticators for at least the main molecular subtypes of the tumor, luminal, basal, and double-negative NMIBC.

Based on the recent studies, we narrowed our current research focus on three molecular pathways—immune checkpoint axis PD1/PD-L1, fibroblast growth factor receptor 3 expression status, and tumor-dependent miRs. The prognostic role of PD-L1 for bladder cancer progression and the treatment success of immune checkpoint inhibitors have been rigorously evaluated in many studies [7,8,9,10,11,12]. In our previous study, we demonstrated that PD-L1 expression level correlated with RFS in relapsed aggressive non-muscular invasive bladder carcinoma of the luminal molecular subtype (GATA3-expressing NMIBC) [12,25]. Kang et al. showed that the *FGFR3* expression level and its gene mutation status associated it with survival in primary pT1 bladder cancer [19]. At the same time, a research team led by Neuzillet demonstrated a link between *FGFR3* mutations, but not expression, and better survival in NMIBC [20]. Meaning of cell-free urine miR-145 and -200a level as marks of emerging bladder cancer was highlighted by Yun [16], whereas many tumor-associated miRs also had predictive value. Having considered the aforementioned, we wondered how relapse-free survival of patients with luminal primary NMIBC might depend on PD-L1 expression, *FGFR3* expression, *FGFR3* gene hotspot mutation status, and tumor-associated miRs-145 and -200a assessed in complex.

We found out that the more aggressive primary luminal tumor also expressed PD-L1 higher than the less aggressive form. Obtained data corresponded with our previous results [12,25]. Contrast data were received during assessment of *FGFR3* expression. High grade primary luminal bladder cancer was associated with low *FGFR3* expression, and vice versa. It should be underlined that we found no correlation between the *FGFR3* gene mutation rate and the receptor expression, despite the mutations intergroup significant differences between high and less aggressive primary tumors. The fact was disputable with previous observations [20]. At the same time, prior results reflected all of the NMIBC population without taking into consideration its molecular heterogeneity. Probably, an increase in the number of observations would allow to get more definite results. We found out that tumor-related miR-200a expression did not vary in high and low grade primary luminal bladder cancer; hence, the molecule had no further prognostic value. In contrast, we detected reciprocal PD-L1-like tumor-related miR-145 expression in study groups. Therefore, not only cell-free but the intracellular level of miR-145 might be considered as a potential predictive biomolecule. We also found out the inverse relationship between miR-145 expression and *FGFR3* regulation in study subgroups. To date, there are no clear proofs of miR-145 to target *FGFR3* expression [26], but the revealed correlation needs further investigation. It might be of great importance taking into consideration Zhu and co-authors recent findings of a positive feedback loop for promoting PD-L1 expression in human bladder cancer cells via the ATG7/autophagy/FOXO3A/miR-145 axis [27]. Beside intergroup differences, it was of great importance to evaluate the markers prognostic value for patients’ survival. It turned out that low *FGFR3* expression in combination with high PD-L1+ status predicted bad survival of patients with primary luminal non-invasive bladder cancer. Obtained data may be used for developing promising prognosticators in oncology. Further observations should be done to clear the predictive value of well-known and novel molecules in basal and double-negative NMIBC with and without p53 mutations, which will be a focus of our subsequent research.

## 4. Materials and Methods

### 4.1. Ethic Procedures

We designed the study in accordance with all requirements to protection of patients’ rights and confidentiality. Study Protocol was reviewed and approved by Ethic Committee of Sechenov University at the Committee Board meeting on 16 September 2019 (Approval No. 9; Rev. No 16/09-1-2019) and Scientific Board of Bio-Ethic Commission of National Research Medical Center of Radiology (Approval No. 12, 2 October 2019; Rev. No. 02/10-2019-E).

### 4.2. Study Population and Follow-Up Protocol

Twenty patients with confirmed luminal molecular subtype type of non-muscular invasive bladder carcinoma who underwent bladder intervention (endoscopic biopsy or transurethral resection) at Oncology Clinic of Sechenov University or Urology Clinical Hospital of National Research Medical Center of Radiology between January 2014 and May 2015 were enrolled in the study. Informed voluntarily consent was given by all the study participants that met the eligibility criteria as follows: (1) age of 18 years or more at the time of the diagnosis; (2) luminal molecular subtype of primary diagnosed non-muscular invasive urothelial carcinoma; (3) available both no longer than 3 year-frozen neoplasm specimens for real-time PCR and formalin-fixed tumor tissue samples for IHC testing. Patients with primary invasive bladder carcinoma, basal, and double-negative NMIBCs were excluded from the study. Before intervention and tissue samples collection, urine of each patient was examined for possible infection. All patients with urinary infection were excluded from the study.

Table A1 summarizes essential participants’ gender data and also contains clinical and morphological features of diagnosed tumors. Eleven males with average age 56.72 ± 3.1 and nine females whose age averaged at 57.55 ± 2.6 formed the study population. For each case, histologic diagnosis was determined along with assessment of individual risk for the tumor recurrence and progression in accordance with European Organization for Research and Treatment of Cancer (EORTC) risk tables [28] and World Health Organization (WHO) classification [29]. Bladder tumors histologically presented by 18 urothelial papillary carcinomas and 2 micropapillary cancers were designated into low grade (12) and high-grade (8) malignancies.

Intervention implemented and surveillance programs were designed for patients in dependence on the lesion stage, grade, and risk. Participants with low grade NMIBC were treated by intravesical Mitomycin 40 mg weekly for six weeks with following cystoscopy and urine cytology at three months after intervention and subsequent observation nine months later if negative. In this subgroup of patients, diagnostic cystoscopy was carried out once a year in years 4–5 of surveillance. Intervention in high grade NMIBC included immunotherapy of Bacillus Calmette-Guerin (BCG) intravesically, with 100 mg per instillation in the same regimen as mentioned above for Mitomycin. Invasive bladder observations in this subgroup were carried quarterly for years 1 and 2 after primary diagnosis, and thereafter twice a year up to five years if throughout the period relapses did not occur. Relapse-free survival (RFS) was defined as the time from first diagnosis of urothelial carcinoma as the primary tumor date to the date of the first documented tumor relapse, or death due to any cause, whichever occurs first.

### 4.3. Tissue Sample Preparation and Processing

All extracted tumor specimens no longer than 5–7 min after intervention were divided into three equal parts. The first one underwent histologic tissue processing resulting in formalin-fixed and paraffin-embedded blocks for further histologic examination. The other parts were immediately frozen at −86 °C. Tissue freezing conditions met basic requirements for biological samples preserving, which would ensure equal diagnostic quality of all samples [30]. One of two frozen samples was designated to miRNAs, detecting *FGFR3* mutations and gene expression determination. The other frozen fragment of tumor tissue sample underwent immunohistochemical testing for GATA3, KRT5/6, and PD-L1 expression. The molecular panel was chosen for the purpose of multifactorial analysis in accordance with the high prognostic value of the biomarkers as survival predictors.

### 4.4. DNA and RNA Isolation

Having been thawed, fragments of freshly frozen tumor tissues were homogenized in 600 µL of RLT Plus buffer solution (Qiagen, Hilden, Germany) with 1% beta-mercaptoethanol with the TissueLyser LT homogenizer (Qiagen, Hilden, Germany) using Lysing Matrix A tubes (MP Biomedicals, Irvine, CA, USA). Nucleic acids were isolated using the AllPrep DNA/RNA/miRNA Universal Kit (Qiagen, Hilden, Germany) according to the manufacturer’s recommendations. DNA and RNA concentrations were measured with a Qubit 4 fluorimeter (“Thermo Fisher Scientific”, Waltham, MA, USA) using, respectively, the Qubit dsDNA HS Assay and Qubit RNA BR Assay kits (“Thermo Fisher Scientific”, Waltham, MA, USA).

### 4.5. Reverse Transcription

For every sample, 100 ng of total RNA was mixed with 20 pm of a reverse transcription primer oligonucleotide (random decamers were used for *FGFR3* and ACTB expression, and specific oligonucleotide primers were used for microRNAs, listed below) in 9 µL and incubated at 70 °C for 2 min, and then they were chilled on ice. Reverse transcription was performed at 42 °C for 30 min using the MMLV RT kit (Evrogen, Moscow, Russia) according to the manufacturer’s recommendations. Reverse transcription was then stopped by inactivating reverse transcriptase by incubating the reaction mixture at 70 °C for 10 min. Obtained cDNA was 10-fold diluted and used for RNA expression analyses using real-time PCR experiments.

### 4.6. Real-Time Polymerase Chain Reaction (PCR)

Real-time PCR experiments were performed using a DTPrime amplifier (DNA technology, Russia). For *FGFR3* and *ACTB* expression, we used qPCRmix-HS SYBR (Evrogen, Moscow, Russia) according to the manufacturer’s recommendations. A measure of 1.5 µL of cDNA solution and 4 pm of each PCR primer were amplified in 25 µL using the following protocol: (1) DNA denaturation at 95 °C for 2 min; (2) 45 cycles of the following: DNA denaturation at 95 °C for 10 s, primer annealing at 67 °C for 3 s, and elongation at 72 °C for 18 s; (3) melting curve analysis.

The following oligonucleotide primers were used for FGFR3: FGFR3RT-F, 5′-CCCAAATGGGAGCTGTCTCG-3′; FGFR3RT-R, 5′-CATCTCAGACACCAGGTCCG-3; for ACTB: b-act-for, 5′-GAGCGGGAAATCGTGCGTGACATT-3′; b-act-rev; 5′-GATGGAGTTGAAGGTAGTTTCGTG-3′.

For microRNAs and for RNU6-1, we used qPCRmix-HS (Evrogen, Moscow, Russia) according to the manufacturer’s recommendations. A measure of 1.5 µL of cDNA solution, 4 pm of each PCR primer, and 3 pm of dual fluorescently labelled detection probe were amplified in 25 µL using the following protocol: (1) DNA denaturation at 94 °C for 2 min; (2) 50 cycles of the following: DNA denaturation at 94 °C for 10 s, and primer annealing and elongation at 53 °C for 20 s; (3) storage at 4 °C.

The following oligonucleotide primers were used: for microRNA-200a: reverse transcription primer *200a-RT*, 5′-*GTCGTGTCTGAGGCTCACTGAGACCTATTCGCACCTCGACACGACACATCGTT*-3′; forward primer *200a-Fw*, 5′-*CCAGCTAACACTGTCTGGT*-3′; reverse primer *UR-3*, 5′-*CTGAGGCTCACTGAGACCT*-3′; for microRNA-145: reverse transcription primer *145-RT*, 5′-*GTCGTGTCTGAGGCTCACTGAGACCTATTCGCACCTGACACGACAGGGATTC*-3′; forward primer *145-FW*, 5′-*CCACAGTCCAGTTTTCCCAG*-3′; reverse primer UR-3 (see above); for *U6* snRNA: reverse transcription primer *U6-RT*, 5′-*GTCGTGTCTGAGGCTGACTGAGACCTATTCGCACCTGACACGACGGCCATGC*-3′; forward primer *U6-Fw*, 5′-*GGCCGCATACAGAGAAGATTA*-3′; reverse primer *U6-Rv*, 5′-*CTGAGGCTGACTGAGACCT*-3′.

The following fluorescently labelled probes were used for transcript detection: for microRNA-200a: *200a-Pb*, 5′-*(R6G)-ATTCGCACC(T-BHQ1)CGACACGACACATCGTT-p*-3′; for microRNA-145: *Pb-145*, 5′-*(R6G)-ATTCGCACC(T-BHQ1)GACACGACAGGGATTC-p*-3′; for U6 snRNA: *U6-Pb*, 5′-*(R6G)-ATTCGCACC(T-BHQ1)GACACGACGGCCATGC*-p-3′.

### 4.7. Detection of FGFR3 Gene Hotspot Mutations

We detected mutations in codons 248 and 249. For this, we PCR-amplified the 7th exon of FGFR3 using 100 ng of genomic DNA, qPCRmix-HS (Evrogen, Moscow, Russia), and 10 pm of the specific primer oligonucleotides (*FGFR3-7F*, 5′-*AGTGGCGGTGGTGGTGAGGGAG*-3′; *FGFR3-7R*, 5′-*ACCTTGAGCACGGTAACGTAGGGTGT*-3′) by the following protocol: (1) DNA denaturation at 95 °C for 3 min; (2) 32 cycles of the following: DNA denaturation at 95 °C for 20 s, primer annealing at 70 °C for 20 s, and elongation at 72 °C for 20 s; (3) storage at 4 °C. PCR products were purified by agarose electrophoresis and the column purification kit Cleanup Standard (Evrogen, Moscow, Russia). Purified PCR products were Sanger-sequenced at Evrogen (Moscow, Russia) using (separately) both *FGFR3-7F* and *FGFR3-7R* primers. Sequencing results were analyzed using Chromas 2.6.6 software (“Technelysium”, Australia). We used non-modified oligonucleotide primers purchased at Evrogen (Moscow, Russia) and fluorescently labeled oligonucleotide probes purchased at DNA-Synthesis (Russia).

### 4.8. Immunohistochemistry (IHC)

We used an immunohistochemical method for two main purposes. First of all, we determined GATA3 and KRT5/6 expression in tumor slices for triage and the identification of a luminal subtype of primary NMIBC, while the valuation of PD-L1 expression level was the other goal of IHC testing (Figure 1). For this, 4-µm-thick sections of tumor tissue were used. Tissue slices were fixed in 10% formalin, and then were dehydrated and embedded in paraffin using an automated regimen of histological processing on the Spin vacuum tissue processor STP250-V (“Histo-Line Laboratories Srl”, Milan, Italy).

To analyze cell expression of the transcription factor encoded by GATA3, we used a monoclonal antibody against human GATA3 (HG3-31 clone, dilution, 1:100; Santa Cruz Biotechnology Inc., Santa Cruz, CA, USA). The expression level of cytokeratin 5 and 6 (CR5/6) was assessed in tumor sections stained by a monoclonal antibody against human CR5/6 (D5/16B4 clone, 1:50 dilution, Dako, Denmark). Luminal molecular subtype of urothelial carcinoma was determined in accordance with Wang et al. [31] and Lerner et al. [32] as a >80% cut-off for GATA3 positive nuclear staining along with low or undetectable CR 5/6 cellular cytoplasm staining.

We used the Ventana PD-L1 Assay (SP263) and the OptiView DAB IHC Detection Kit (Cat. No. 760-700/06396500001) with signal amplification (Ventana Medical Systems, Inc., Tucson, AZ, USA) to evaluate the PD-L1 expression level in urothelial carcinoma sections. Negative control was stained by primary Ventana Rabbit Monoclonal Negative Control antibody (Cat. No. 790-4795/06683380001). In each reaction cycle, tonsils tissue specimens served as a positive control. To set up a reaction with the SP263 assay, after the dewaxing and unmasking antigens, rabbit monoclonal antibody PD-L1 (SP263) in working dilution was applied to prepared tumor sections. We used the Ventana BenchMark ULTRA ICH stainer (Ventana Medical Systems Inc., Tucson, AZ, USA) in accordance with the manufacturer’s guidelines for the reaction automated processing [33]. We scored the percentage of PD-L1-reacted cells in five randomly PC-selected high-power fields under light microscope with 500-folds magnification. High PD-L1 status was classified if ≥25% of tumor cells (TC) and/or ≥25% of immune cells (if they represented more than 1% of total cell population) displayed membrane PD-L1 positivity. Cellular membrane staining positivity scored between 0 and 25% for aforementioned cell populations was classified as Low PD-L1+ status. Absence of membrane positive staining appropriated a negative PD-L1 status.

### 4.9. Statistical Analysis

Data were processed and handled using the SPSS statistical software program, version 22.0 (SPSS, Inc., Chicago, IL, USA). Independent *t* test and analysis of variance (ANOVA) were used to compare the expression of PD-L1, miR-200a, miR-145, and *FGFR3* between groups. The Mann–Whitney U test was used to compare the *FGFR3* gene mutations between groups. The Kaplan–Meier method was used to estimate the time to first relapse and relapse-free survival, and differences were assessed using log-rank statistics. The prognostic values of the tumor grade, PD-L1, miR-200a, miR-145, and *FGFR3* expressions were analyzed by univariable Cox proportional hazard regression models.

## 5. Conclusions

Tumor grade in association with PD-L1 and *FGFR3* expression can be considered a complex predictor for primary luminal NMIBC progression.

## Figures and Tables

**Table 1:** Univariable Cox regression models for prediction of recurrence in primary luminal NMIBC.

Variables	HR	95% CI	*p*-Value
Tumor grade	571.72	11.03–2.96	0.002
PD-L1 expression	2.33	0.92–1.92	0.012
miR-200a expression	0.98	0.95–1.01	0.40
miR-145 expression	0.99	0.93–1.06	0.96
*FGFR3* expression	0.08	0.17–0.42	0.003
*FGFR3* gene mutations	1.46	0.35–6.00	0.593

## Abbreviations

BCG	Bacillus Calmette-Guerin
EORTC	European Organization for Research and Treatment of Cancer
FGFR3	Fibroblast growth factor receptor 3
GATA3	Transcription factor encoded by GATA3 gene
IHC	Immunohistochemistry
CR5/6	Cytokeratin 5 and 6
MIBC	Muscular-invasive bladder cancer
miR	microRNA
NMIBC	Non-muscular invasive bladder cancer
PCR	Polymerase chain reaction
PD1	Programmed death receptor 1
PD-L1	Programmed death receptor ligand 1
RFS	Relapse-free survival
TUR	Transurethral resection

## Appendix A

**Table A1:** Tumor-related characteristics of the study participants.

Patient	Tumor Grade and Stage	Gender	Age, Years	Tumor Histology
1	T1, High grade	Male	54	UPC
2	T1, Low grade	Male	57	UPC
3	T1, High grade	Female	61	MC
4	T1, Low grade	Male	60	UPC
5	T1, Low grade	Female	51	UPC
6	T1, Low grade	Female	64	UPC
7	T1, High grade	Female	67	UPC
8	T1, High grade	Male	59	UPC
9	T1, Low grade	Female	53	UPC
10	T1, High grade	Male	49	MP
11	T1, Low grade	Male	58	UPC
12	T1, Low grade	Female	50	UPC
13	T1, Low grade	Male	55	UPC
14	T1, Low grade	Male	48	UPC
15	T1, Low grade	Male	63	UPC
16	T1, High grade	Female	70	UPC
17	T1, High grade	Male	69	UPC
18	T1, Low grade	Female	47	UPC
19	T1, Low grade	Female	55	UPC
20	T1, High grade	Male	52	UPC
Total	Low grade (12), High grade (8)	Male (11), Female (9)	Average (Mean ± SD) 57.1 ± 2.8	UPC (18) MC (2)

UPC—urothelial papillary carcinoma; MC—micropapillary carcinoma.
